# Signet ring cell carcinoma of colonic origin in a 13-year-old child from remote setting: a case report

**DOI:** 10.1186/s13256-026-06286-3

**Published:** 2026-07-16

**Authors:** Dawit Admasu Woldesenbet, Adugnaw Atnafu Atalay, Atsede Birhanu Worku, Gebre Hailu Dejen, Zeru Seyoum Wondimagegn, Zenebe Daniel Getachew, Abayneh Tunta Boye

**Affiliations:** Department of Surgery, School of Medicine, College of Health Sciences, Woldia University, Woldia, Gomata, Ethiopia

**Keywords:** Right colonic adenocarcinoma, Signet cell carcinoma, Immunohistochemistry

## Abstract

**Introduction:**

Signet ring cell carcinoma (SRCC) is a distinct and aggressive subtype of adenocarcinoma that can arise throughout the tubular gastrointestinal tract, with particularly rare occurrence in the colon, especially in the pediatric population.

**Case presentation:**

A 13-year-old girl from the Amhara region of Ethiopia presented with a long standing history of intermittent, crampy abdominal pain, accompanied by loss of appetite, unquantified weight loss, and easy fatigability. Two months prior to admission, her symptoms worsened, and over the course of two days, the pain became persistent and was accompanied by frequent bilious vomiting and failure to pass feces and flatus. On physical examination the patient was restless, tachycardic and in pain. Her abdomen was grossly distended and tympanic on percussion. DRE revealed an empty rectum. With the presumptive diagnosis of ileocolic intussusception the patient underwent further investigations. CBC revealed WBC count of 12,100/µL with neutrophil predominance (76%), hemoglobin 14.2 g/dl, and a platelet count of 442 × 10^3^/µL. An erect abdominal X-ray showed centrally located dilated bowl loops with multiple air-fluid levels. Abdominal ultrasound revealed a concentric bowel wall thickening involving the cecum and ascending colon, measuring approximately 12 mm in thickness, along with multiple adjacent enlarged paracolic and mesenteric lymph nodes. After preoperative optimization, administration of prophylactic antibiotics, and obtaining informed consent, the patient underwent exploratory laparotomy. Intraoperatively, a circumferentially thickened cecal mass causing luminal obstruction was identified, with marked proximal small bowel dilatation and multiple mesenteric lymphadenopathies.

**Discussion:**

Colorectal cancer accounts for approximately 10% of all cancer cases worldwide and remains a leading cause of cancer-related mortality. SRCC of the colon is a rare and aggressive histological subtype characterized by poor prognosis and distinct biological behavior.

**Conclusion:**

Colorectal carcinoma in pediatric age group, although rare, may present with nonspecific and atypical symptoms, leading to delayed diagnosis. Therefore, colonic malignancy should be considered in the differential diagnosis of children presenting with chronic, unexplained abdominal pain and bowel obstruction symptoms, as early detection and management of the disease have a pivotal role in the prognosis.

## Introduction

Colorectal cancer (CRC) remains one of the leading causes of cancer-related mortality worldwide, ranking among the most common malignancies and a major contributor to global cancer burden [1, 2]. Signet ring cell carcinoma (SRCC) is a distinct and rare histological subtype of adenocarcinoma that can arise throughout the tubular gastrointestinal tract [3]. It is characterized by abundant intracellular mucin displacing the nucleus to the periphery, and a diagnosis of SRCC is established when more than 50% of tumor cells exhibit this morphology [4].

CRC is uncommon in the pediatric and young adult population. The prevalence among individuals under 20 years of age is estimated to be approximately 0.2%. Although the Surveillance, Epidemiology, and End Results (SEER) program reports over 145,000 new CRC cases annually, only a small fraction—approximately 290 cases—occur in children and young adults. Despite an overall decline in CRC incidence in the general population, likely due to widespread screening programs such as colonoscopy, recent data suggest a concerning rise in incidence among younger individuals [2, 5].

SRCC of the colon is particularly rare, accounting for approximately 0.6–1.1% of all colorectal cancers [6]. It typically presents at an advanced stage, largely due to its infiltrative and endophytic growth pattern, which delays early detection [7]. Although the majority of cases are sporadic, an association with inflammatory bowel disease has been reported in approximately 14% of patients [8].

Clinically and biologically, SRCC differs significantly from conventional colorectal adenocarcinoma. It tends to occur at a younger age, with a predilection for the proximal (right-sided) colon, and is frequently associated with advanced disease at presentation. Histologically, it demonstrates high-grade features, extensive intramural spread, and a strong propensity for lymph node involvement and peritoneal metastasis [9]. These characteristics contribute to its aggressive behavior and poorer prognosis compared to other colorectal cancer subtypes.

## Case presentation

A 13-year-old girl from the Amhara region of Ethiopia presented with a long standing history of intermittent, crampy abdominal pain, accompanied by loss of appetite, unquantified weight loss, and easy fatigability. Two months prior to admission, her symptoms worsened, and over the course of two days, the pain became persistent and was accompanied by frequent bilious vomiting and failure to pass faces and flatus. She had no personal and family history of similar illness. She was an active primary school student with no know medical and surgical conditions and was fully vaccinated for her age.

On physical examination the patient was restless, tachycardic and in pain. Her abdomen was grossly distended and tympanic on percussion. Digital rectal examination (DRE) revealed an empty rectum.

**Fig. 1:**
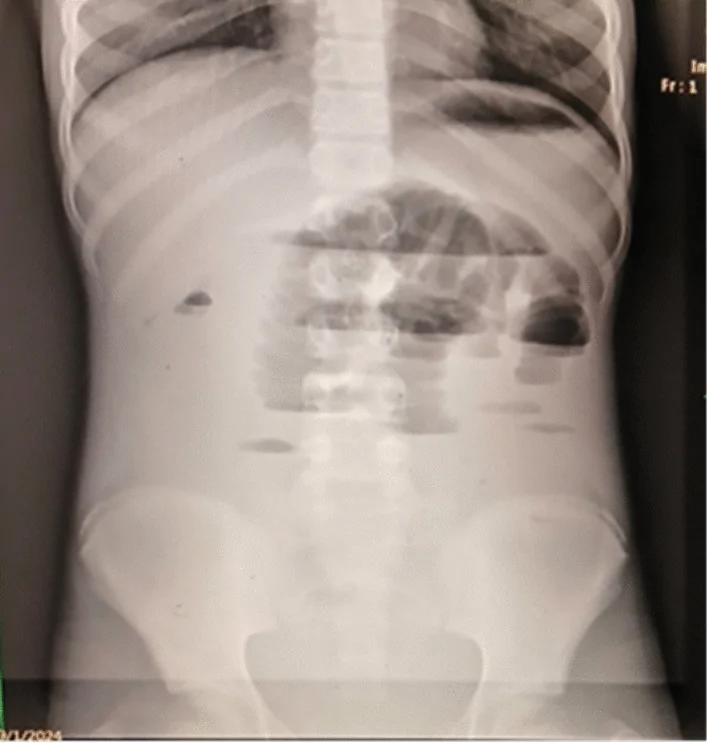
Erect abdominal X-ray of patient

With the presumptive diagnosis of ileocolic intussusception the patient underwent further investigations. Complete blood count (CBC) revealed White blood cell count (WBC) count of 12,100/µL with neutrophil predominance (76%), hemoglobin 14.2 g/dl, and a platelet count of 442 × 10^3^/µL. An erect abdominal X-ray showed centrally located dilated bowl loops with multiple air-fluid levels. Abdominal ultrasound revealed a concentric bowel wall thickening involving the cecum and ascending colon, measuring approximately 12 mm in thickness, along with multiple adjacent enlarged paracolic and mesenteric lymph nodes (Fig. 1).

**Fig. 2:**
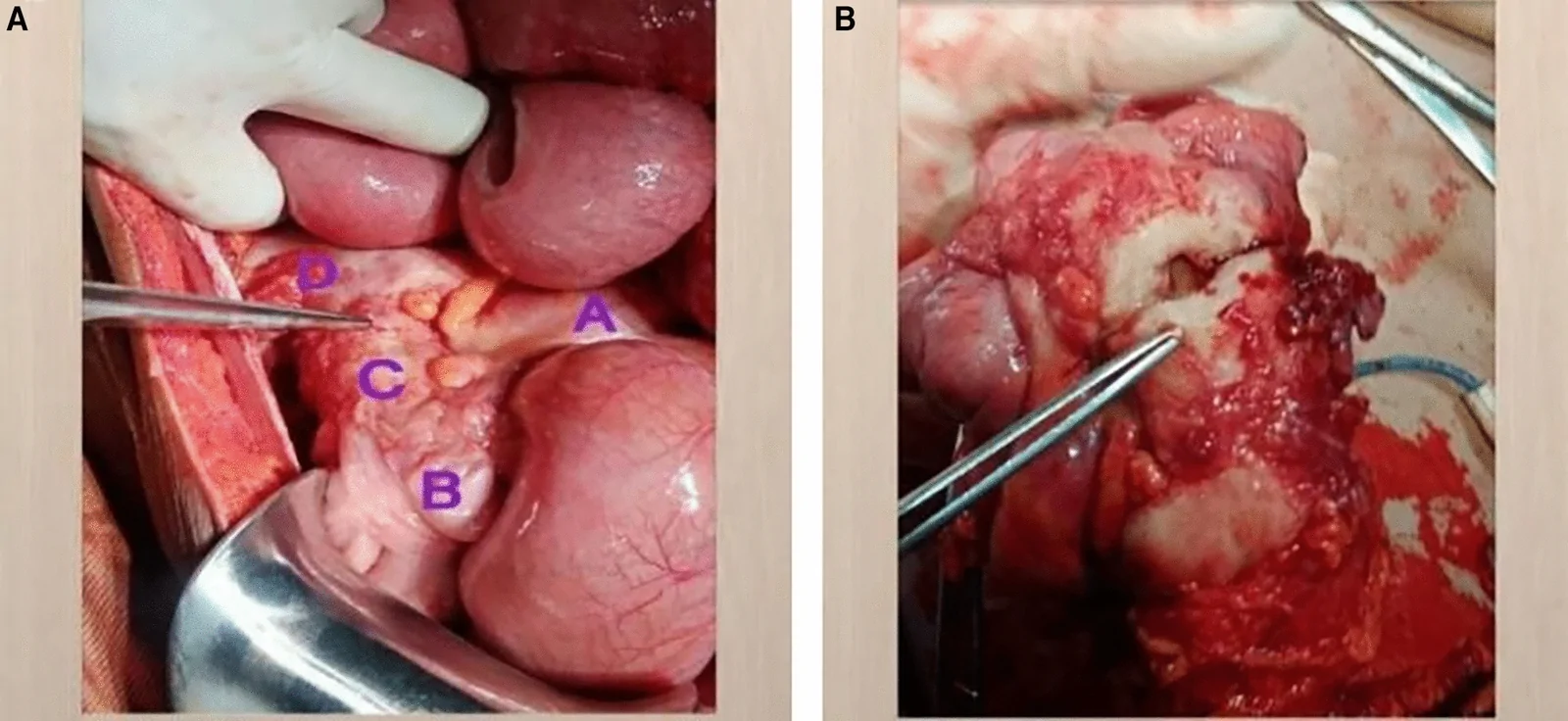
**A**: Right colon cut edge view (intraoperative image) (A=Terminal ileum B=Cecum C=Obstructing cecal mass D=Ascending colon) **B**: Circumferentially thickened obstructing cecal mass (intraoperative image)

After appropriate preoperative optimization with intravenous (IV) fluid and prophylactic antibiotics, and obtaining informed consent from the family, the patient was taken to operating room. Exploratory laparotomy via a long vertical midline incision revealed a circumferentially thickened cecal mass causing luminal obstruction, with marked proximal small bowel dilatation and multiple mesenteric lymphadenopathies (Fig. 2). The remaining bowel segments, liver, and other intra-abdominal organs appeared grossly normal.

A standard right hemicolectomy was performed following mobilization of the distal ilium, cecum and right colon. The resected specimen was sent for histopathologic and immunohistochemistry (IHC) analysis.

The postoperative course was uneventful, and the patient was discharged on the fifth postoperative day after assurance of resumption of bowel function and stable vital signs. At the two-week follow-up, she was clinically well, with a healed midline surgical wound.

**Fig. 3:**
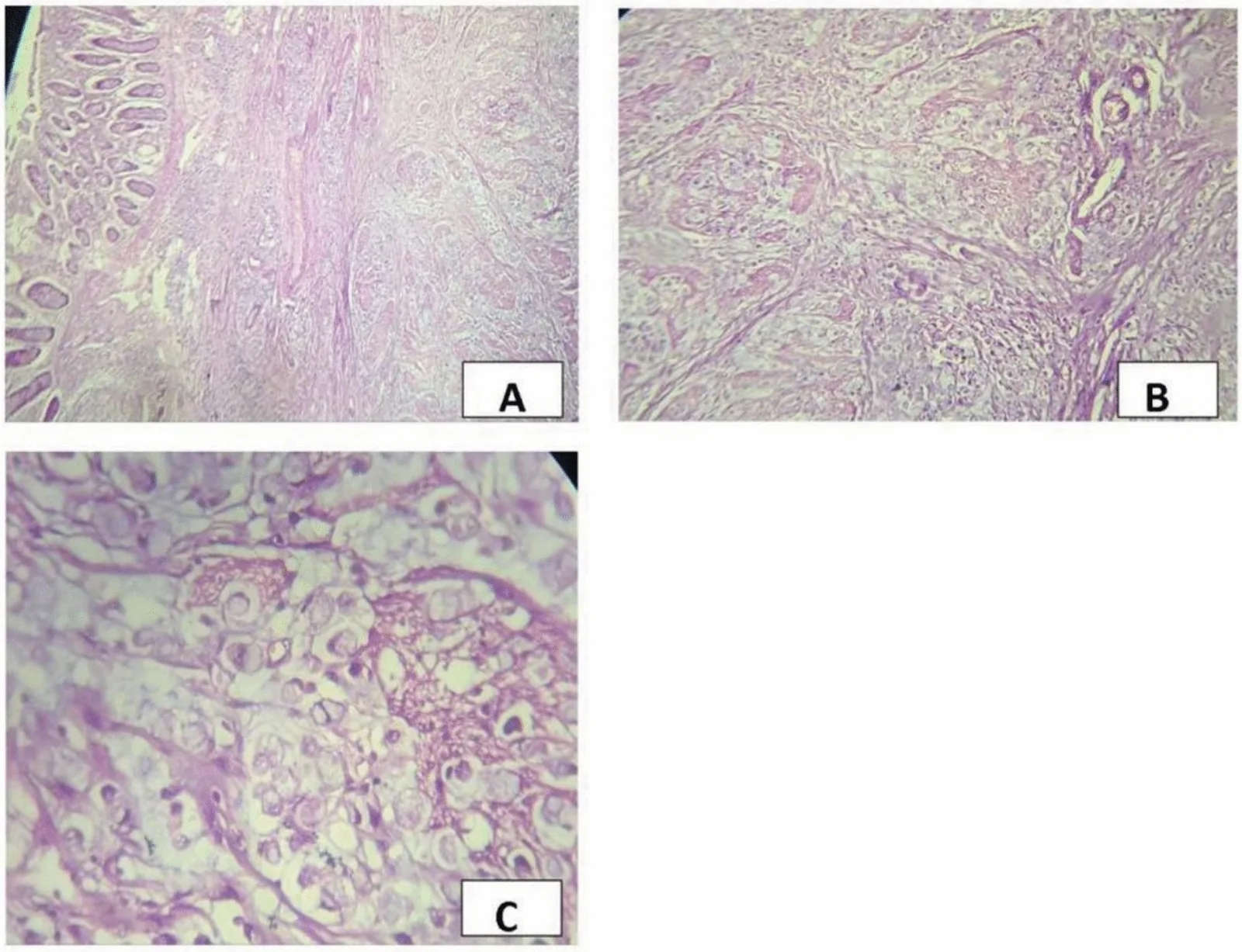
Photomicrograph of sample from colon showing solid sheets of signet ring cells with eccentrically located nuclei and abundant mucus filled cytoplasm infiltrating into the muscularis layer (A=initial scanning lower power field view (10X), B=40X view, C=high power field view (100X))

**Fig. 4:**
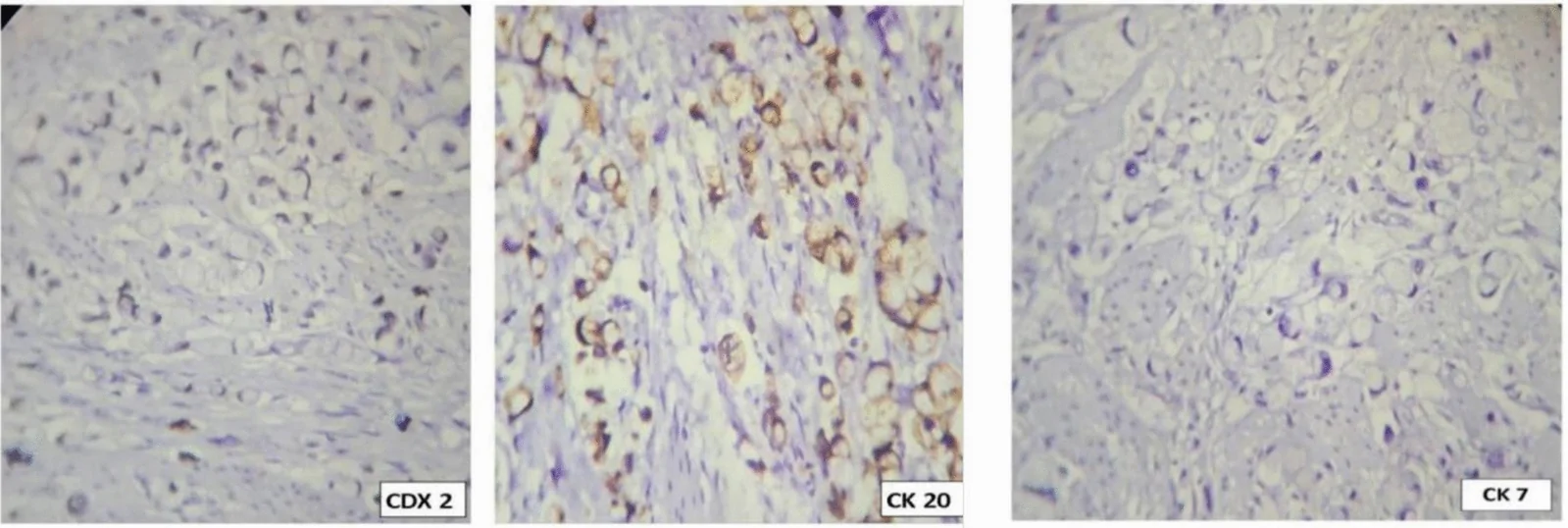
Immunohistochemistry of a sample from colon of 13-year-old child

Histopathological examination revealed right colonic adenocarcinoma with predominantly signet ring cell features. IHC analysis further confirmed the diagnosis of primary SRCC of colonic origin (Figs. 3 and 4).

Due to the unavailability of computed tomography (CT) scan and colonoscopy at our institution, the patient was referred to a higher center for further evaluation and management. Subsequent colonoscopy showed no additional colonic lesions, and CT imaging revealed no evidence of local or distant metastasis.

### Investigation results

#### Preoperative CBC

Leukocytosis was noted with WBC count of 12.1 × 103/µL and neutrophil predominance (76%). Hemoglobin level was 14.2 g/dL, and platelet count was 442 × 103/µL.

#### Postoperative CBC

The WBC count increased to 14.6 × 103/µL with marked neutrophil predominance (90%). Hemoglobin decreased to 11 g/dL, while the platelet count was 368 × 103/µL.

#### Erect abdominal X-ray

Imaging demonstrated centrally located dilated small bowel loops measuring up to 6.5 cm in diameter, with absence of rectal gas shadow (Fig. 1).

#### Abdominal ultrasound

There are multiple fluid filled dilated small bowel loops the widest measuring 4.5 cm in diameter. There is fro and back peristaltic movement suggesting mechanical obstruction. There is telescoping of bowel loops on itself forming concentric rings in the right iliac fossa measuring 3.5 cm in length and 2.3 cm in Antero-posterior (AP) diameter. The ascending colon just proximal to this telescoping is circumferentially thickened and has lost bowel signature. It measures up to 12 mm. There are multiple enlarged hypoechoic mesenteric lymph nodes in the right iliac fossa. There is minimal anechoic free intraperitoneal fluid. There is no organomegaly.

#### Microscopic description

- Procedure:* colectomy*
- Site of tumor:* colon*
- Histologic type/diagnosis:* kindly refer ADU 284*
- Immunohistochemistry:* selected panel*
- Monoclonal antibody used:* CK20, CK7 and CDX2*
- Interpretation: cells are diffusely positive for CK20 and CDX2 and negative for CK7

## Discussion

About 10% of all cancer cases are colorectal cancer, making it the second most common cause of cancer-related deaths globally and the third most common malignancy overall [10]. The right side of the colon is the most common location for signet cell carcinoma, although approximately 20% of cases have been reported in the rectum. Although the exact cause is still unclear, colorectal SRCC has a poorer prognosis compared to mucinous adenocarcinoma. The potential carcinogenic pathway includes inflammation–metaplasia–dysplasia–carcinoma sequence [11]. Colonic SRCC is an uncommon and highly aggressive form of adenocarcinoma, which is characterized by distinct molecular and biologic behavior and generally poor prognosis [12].

We report a case of colonic SRCC in a 13-year-old girl, highlighting its early onset and the recognized female predominance in younger patients. SRCC predominantly affects individuals under 40 years of age, and younger patients are reported to be approximately four times more likely to develop SRCC compared to older adults [13, 14]. In our patient, the presenting symptoms included crampy abdominal pain, anorexia, and weight loss. This clinical presentation is consistent with previously reported nonspecific symptoms such as altered bowel habits, abdominal pain, rectal bleeding, and unintentional weight loss [14]. Due to its nonspecific and late-onset clinical manifestations, SRCC is often diagnosed at an advanced stage with lymph node involvement or distant metastasis, which limits surgical options and contributes to poor prognosis.

Histopathological examination in our case revealed solid sheets of signet ring cells characterized by eccentrically placed nuclei and abundant intracellular mucin infiltrating the muscularis layer. SRCC is distinguished histologically from conventional colorectal adenocarcinoma by the presence of > 50% intracytoplasmic mucin, which displaces the nucleus peripherally, creating the characteristic signet ring appearance. Molecularly, SRCC has been associated with a higher frequency of BRAF mutations, a relatively lower incidence of KRAS mutations, and a higher rate of microsatellite instability (MSI) compared to conventional adenocarcinoma [15].

In the present case, intraoperative findings included a circumferentially thickened cecal mass causing luminal obstruction, with proximal small bowel dilatation and multiple mesenteric lymphadenopathies. A standard right hemicolectomy was performed. Recent evidence suggests that surgical resection plays a crucial role in selected cases of SRCC, including advanced disease, and may contribute to improved overall survival [16].

Immunohistochemical analysis in our case demonstrated diffuse positivity for CK20 and CDX2, while CK7 was negative. This immunoprofile is consistent with SRCC of gastrointestinal origin, as most cases typically exhibit CK20 and CDX2 positivity with CK7 negativity [17, 18].

## Conclusion

Colorectal carcinoma in pediatric age group, although rare, may present with nonspecific and atypical symptoms, leading to delayed diagnosis. Therefore, colonic malignancy should be considered in the differential diagnosis of children presenting with chronic, unexplained abdominal pain and bowel obstruction symptoms, as early detection and management of the disease have a pivotal role in the prognosis.

**Table:** Timeline of events

Date	Event and respective action taken
20/02/2023	The patient’s symptoms progressively worsened and became persistent
22/02/2023 (morning)	The patient presented to the emergency department with worsening abdominal pain, accompanied by bilious vomiting and failure to pass feces and flatus
22/02/2023 (mid-day)	The patient was taken to the operating room, where exploratory laparotomy and a standard right hemicolectomy were performed. The resected specimen was subsequently sent for histopathological and IHC analysis
27/02/2023	The patient was discharged home on the fifth postoperative day
13/03/2023	First follow-up visit (second postoperative week), the patient was comfortable and had no complaints. Histopathological examination of the resected specimen revealed right colonic adenocarcinoma with predominantly signet ring cell features
03/04/2023	Second follow-up visit (fifth postoperative week), the patient was comfortable and had no complaints. IHC analysis confirmed the diagnosis of SRCC of colonic origin
24/04/2023	Third follow-up visit (eighth postoperative week), the patient was comfortable and had no complaints. Further evaluation with colonoscopy and computed tomography (CT) scan revealed no identifiable colonic lesions and no evidence of local or distant metastasis

## Data Availability

Throughout the course of the patient care starting from the preoperative evaluation up to the intraoperative and postoperative follow-ups, the patient data and important investigation findings had been kept and are available to address any further inquiry.

## Abbreviations

BP: Blood pressure
BRAF: B-Rapidly accelerated fibrosarcoma
CBC: Complete blood count
DRE: Digital rectal examination
GCS: Glasgow Coma Scale
IBD: Inflammatory bowel disease
ICU: Intensive care unit
IHC: Immunohistochemistry
IV: Intravenous
KRAS: Kirsten rat sarcoma
PR: Pulse rate
RR: Respiratory rate
SRCC: Signet ring cell carcinoma
WBC: White blood cell count
